# Retraction: Genistein Improves 3-NPA-Induced Memory Impairment **in** Ovariectomized Rats: Impact **of** Its Antioxidant, Anti-Inflammatory and Acetylcholinesterase Modulatory Properties

**DOI:** 10.1371/journal.pone.0336073

**Published:** 2025-11-05

**Authors:** The PLOS One Editors

Following the publication of this article [1], concerns were raised for Figs 3, 6, 7, 8 and 9. Specifically, there are regions within the following figure panels which appear similar:

- Fig 3F, 3G
- Fig 6A, 6B, 6D, 6E, 6F, 6G
- Fig 7D, 7E
- Fig 8A, 8B, 8C, 8D, 8E, 8F
- Fig 9A, 9B, 9D, 9G

In light of the above concerns that question the reliability and integrity of these results, the *PLOS One* Editors retract this article. In addition, results presented in Fig 3 of this article [1] appear similar to results presented in Fig 5 of [2, retracted in 3], published later by the same researchers. All authors either did not respond directly or could not be reached.
